# Mitochondrial genomes of African pangolins and insights into evolutionary patterns and phylogeny of the family Manidae

**DOI:** 10.1186/s12864-017-4140-5

**Published:** 2017-09-21

**Authors:** Zelda du Toit, Morné du Plessis, Desiré L. Dalton, Raymond Jansen, J. Paul Grobler, Antoinette Kotzé

**Affiliations:** 1 Department of Genetics, University of the Free State, P.O. Box 339, Bloemfontein, 9300 South Africa; 2 Centre for Conservation Science, National Zoological Gardens of South Africa, P.O. Box 754, Pretoria, 0001 South Africa; 3 Department of Zoology, University of Venda, Thohoyandou, South Africa; 4 Department of Environmental, Water and Earth Sciences, Tshwane University of Technology, Private Bag X680, Pretoria, 0001 South Africa

**Keywords:** Mitochondrial DNA, Phylogenetics, Pholidota, African pangolins

## Abstract

**Background:**

This study used next generation sequencing to generate the mitogenomes of four African pangolin species; Temminck’s ground pangolin (*Smutsia temminckii*), giant ground pangolin (*S. gigantea*), white-bellied pangolin (*Phataginus tricuspis*) and black-bellied pangolin (*P. tetradactyla*).

**Results:**

The results indicate that the mitogenomes of the African pangolins are 16,558 bp for *S. temminckii*, 16,540 bp for *S. gigantea*, 16,649 bp for *P. tetradactyla* and 16,565 bp for *P. tricuspis*. Phylogenetic comparisons of the African pangolins indicated two lineages with high posterior probabilities providing evidence to support the classification of two genera; *Smutsia* and *Phataginus*. The total GC content between African pangolins was observed to be similar between species (36.5% – 37.3%). The most frequent codon was found to be A or C at the 3rd codon position. Significant variations in GC-content and codon usage were observed for several regions between African and Asian pangolin species which may be attributed to mutation pressure and/or natural selection. Lastly, a total of two insertions of 80 bp and 28 bp in size respectively was observed in the control region of the black-bellied pangolin which were absent in the other African pangolin species.

**Conclusions:**

The current study presents reference mitogenomes of all four African pangolin species and thus expands on the current set of reference genomes available for six of the eight extant pangolin species globally and represents the first phylogenetic analysis with six pangolin species using full mitochondrial genomes. Knowledge of full mitochondrial DNA genomes will assist in providing a better understanding on the evolution of pangolins which will be essential for conservation genetic studies.

**Electronic supplementary material:**

The online version of this article (10.1186/s12864-017-4140-5) contains supplementary material, which is available to authorized users.

## Background

Worldwide, the eight extant pangolin species are classified in the order Pholidota which consists of one family, Manidae (Class: Mammalia). The four African species includes Temminck’s ground pangolin (*Smutsia temminckii*), giant ground pangolin (*S. gigantea*), white-bellied pangolin (*Phataginus tricuspis*) and black-bellied pangolin (*P. tetradactyla*) [1–3]. The four Asian species include Philippine pangolin (*Manis culionensis*), Indian pangolin (*M. crassicaudata*), Chinese pangolin (*M. pentadactyla*) and Malayan pangolin (*M. javanica*). All African pangolin species are listed as *Vulnerable* on the International Union for Conservation of Nature (IUCN) Red List of Threatened Species [4–7]. Of the Asian species, two are listed as *Critically Endangered* (Chinese and Malayan pangolin) [8, 9] and two are listed as *Endangered* (Philippine and Indian pangolin) [10, 11]. Pangolins face numerous threats, including habitat destruction [12–14], electrocution [15–17] as well as poaching and illegal trade [18–23]. In 2016, the IUCN voted in support of transferring all eight pangolin species from Appendix II to Appendix I at the Convention on International Trade in Endangered Species of Fauna and Flora (CITES), which was approved at the 17th Conference of Parties (COP17). The listing has resulted in worldwide commercial trade in pangolins being banned as from January 2017 [24, 25]. The taxonomy of pangolins is still under debate, with disagreement regarding the number of genera due to lack of molecular phylogenetic analysis [1, 3, 26–29]. These species have been placed into six genera by Pocock [26]. Other authors have classified all eight extant species of pangolins into a single genus; *Manis* [3, 29, 30]. Corbet and Hill [31] suggested two genera; *Manis* (Asian pangolins) and *Phataginus* (African pangolins) while Koeningswald [32] and Gaudin and Wible [1] proposed three genera; *Manis* (Asian pangolins), *Phataginus* (African tree pangolins) and *Smutsia* (African ground pangolins). Based on osteological characteristics from the entire skeleton [33], three genera were supported, with the first two genera (*Phataginus* and *Smutsia*) forming a monophyletic African clade in the subfamily Smutsiinae [33]. Lastly, four genera have been proposed by McKenna and Bell [28] and Kingdon [34] namely *Manis*, *Smutsia*, *Phataginus* and *Uromanis*. Several authors follow the single genus classification [35–38], however an in-depth taxonomic study of pangolin genera is required in order to clarify this issue.

Mitochondrial DNA (mtDNA) accounts for 1-2% of total DNA content found in mammalian species [39] and is circular, double-stranded and between 14 and 19 kb in length [40]. The vertebrate mitochondrial genome generally consists of 37 genes, specifying 13 proteins, two ribosomal RNAs, 22 tRNAs, and a control region [41]. The control region is non-coding and contains elements that may regulate replication and transcription [42]. Mitochondrial DNA is generally suitable for evolutionary studies due to its high mutation rate, well-structured genome with restricted non-coding DNA sequences and lack of recombination. Several studies have used portions of the mitochondrial genome including the control region (D-loop) [43, 44], cytochrome c oxidase I (*CoxI*) [44, 45], cytochrome B (*Cob*) [44, 46, 47] and 16S ribosomal RNA (16S rRNA) [47] for traceability of confiscated pangolin scales. Whole mitochondrial DNA genomes will however be more informative for phylogenetic analysis [48–53]. To date, full mitochondrial genomes of five pangolin species have been determined including *M. pentadactyla*, *M. javanica*, *S. temminckii*, *P. tetradactyla* and *P. tricuspis* [54–59]. However, two mitogenomes include misidentified Genbank records incorrectly accessioned as *M. pentadactyla* and *P. tetradactyla* that were noted in subsequent studies [58, 60]. Several techniques have been reported to generate whole mitochondrial genomes, however modern techniques such as next generation sequencing (NGS) using 454, Illumina and Ion Torrent technology have simplified and made sequencing mitogenomes from any eukaryotic DNA easier, quicker and more affordable compared to Sanger-based methods [61–63]. The vast suite of Bioinformatics software currently available facilitates the annotation and aids in analyses of large datasets [64].

In general, a quarter of the reads generated by RNA/DNA sequence experiments are from mitochondrial genomes [61, 64–66] which may be attributed to their high copy numbers as well as their high expression levels. Due to the AT richness of mtDNA, as well as it being polyadenylated it can contribute to an increase in poly-A RNA selection [65]. Assembling mitochondrial genomes are significantly less complex than their nuclear genome counterparts as they are smaller in size, and harbour fewer genes [64]. The mitogenomes of two Asian pangolin species (*M. pentadactyla* and *M. javanica*) have been assembled using Illumina HiSeq technology, whereby the authors extracted mitochondrial sequences from nuclear data obtained from NGS techniques [57, 59].

Current phylogenetic assessments of pangolins have been conducted using only two of the four African pangolin species namely; Temminck’s ground pangolin and white-bellied pangolin [56, 58]. In addition, the current genus-level classification of pangolins is still under debate. Thus, in this study we performed next generation sequencing for all four African pangolins using the Illumina HiSeq 2500 in order to reconstruct complete mitochondrial genomes. Here we present the first whole mitochondrial DNA genomes of two of the African pangolin species; the black-bellied pangolin (*P. tetradactyla*) and the giant ground pangolin (*S. gigantea*). In addition, we describe the mitochondrial genome features in order to understand the evolutionary forces shaping the mitochondrial genomes of African pangolins. Lastly, we conduct a phylogenetic assessment in order to provide a genus-level classification of African pangolins.

## Methods

### Sample collection and DNA isolation

This study used six deceased individuals, sampled by the African Pangolin Working Group (APWG) and representing the four African pangolin species. Tissue samples were placed in absolute ethanol and were stored at the National Biobank, National Zoological Gardens of South Africa (NZG), at −80 °C until analysis. The samples were from one black-bellied pangolin (*P. tetradactyla*; MF509825), one white-bellied pangolin (*P. tricuspis*; MF536683), both from Ghana [67]; and three Temminck’s ground pangolins (*S. temminckii*; MF536685–MF536687) from South Africa. In addition, a giant ground pangolin (*S. gigantea*; MF536684) scale sample was included from an illegal seizure. The species identity of samples used in this study was confirmed with Sanger sequencing of the *CoxI* and *Cob* loci which were compared to chain-of-custody voucher specimens available from the NZG species reference database [68] (see http://www.barcodeofwildlife.org). All voucher specimens were verified and identified by an acknowledged authority (Raymond Jansen; African Pangolin Working Group). DNA was isolated using the QIAamp Micro Kit (QIAGEN, Novato, CA, USA) and the respective manufacturers’ protocol for tissue was followed. DNA was quantified on the Qubit 3.0 Fluorometer (Thermo Scientific, Massachusetts, USA). Polymerase Chain Reaction (PCR) amplification and sequencing, to verify species identity, were performed as outlined in Mwale [44].

### Next-generation sequencing and assembly

The products were run on an Illumina HiSeq 2500 (Illumina Incorporated, San Diego, CA, USA) using a rapid run and the TruSeq DNA LT Sample Prep Kit (Illumina Incorporated, San Diego, CA, USA). Data quality was evaluated using FastQC v0.11.2 [69] software, and trimmed and edited through Trimmomatic v0.36 [70] to remove the adapters and poor quality sections. Mitogenomes were assembled in CLC Genomics Workbench v6 (https://www.qiagenbioinformatics.com; CLC Bio, Aarhus, Denmark) using De Novo alignment, with paired reads. Sequence identity of contigs was validated by performing a BLAST search on the National Centre for Biotechnology Information (NCBI) website (http://blast.ncbi.nlm.nih.gov/Blast.cgi).

### Mitogenome annotation and phylogenetic analysis

The mitogenomes were annotated with MITOS v806 [71] and a circular alignment between the six available pangolin species were drawn in Circos v0.69 [72]. The GC content of the four African pangolin mitogenomes were calculated using GPMiner [73] with a sliding window of 300 bp. Arlequin v3.5.1 [74] was used to validate the GC scores obtained for the four mitogenomes using ANOVA analysis and the diagrams were plotted in R v3.3.1 [75].

The mitogenomes generated in this study comprised six animals from four African pangolin species and were combined and aligned with 11 other genomes using MAFFT v7 [76] (Table 1). The mitogenomes of *Acinonyx jubatus* [77], *Crocuta crocuta* [78], *Canis lupus* [79] *and Arctocephalus pusillus* [80] were used as out-groups, as the order Pholidota (pangolins) is reported to be evolutionary closer to carnivorans [56]. The phylogenetic program jModeltest v2.1.7 [81] was used to determine the best fit model of sequence evolution, under the Akaike Information Criterion (AIC) [82], Bayesian Information Criterion (BIC) [83] and Decision Theory Performance-Based Selection (DT) [84]. Partition analysis was also implemented using the program PartitionFinder v2 [85] to determine the best fit models of evolution for the different loci in the dataset. The partition was run using linked branch lengths and a greedy search for the models under the AIC. Phylogenetic analysis was conducted using MrBayes v3.2.6 [86] to infer relationships between the different species using Bayesian Inference (BI). The parameters used for MrBayes were two million generations after which 25% of the trees were discarded as burn-in. A Maximum Likelihood (ML) tree was constructed utilizing PhyML v3 [87] with the same models used for the Bayesian analysis and was run with 10,000 Bootstrap replications. Individual phylogenetic trees, for each loci, were also created with MrBayes v3.2.6 [86] and PhyML v3 [87].

**Table 1:** List of 17 mitogenomes used in the study presented here

Common Name	Scientific Name	Genbank Accession Number	Reference
Cheetah	*Acinonyx jubatus*	AY463959.1	[77]
Spotted Hyena	*Crocuta crocuta*	JF894378.1	[78]
Grey Wolf	*Canis lupus*	KU696410.1	[79]
Brown Fur Seal	*Arctocephalus pusillus*	NC_008417.1	[80]
Chinese Pangolin^a^	*Manis pentadactyla*	JN411577.1	[55]
Chinese Pangolin	*Manis pentadactyla*	KT445978.1	[59]
Malayan Pangolin	*Manis javanica*	KP306515.1	[58]
Malayan Pangolin	*Manis javanica*	KT445979.1	[57]
Black-Bellied Pangolin^b^	*Phataginus tetradactyla*	AJ421454.1	[54]
White-Bellied Pangolin	*Phataginus tricuspis*	KP306514.1	[58]
Temminck’s Ground Pangolin	*Smutsia temminckii*	KP125951.1	[56]
Temminck’s Ground Pangolin	*Smutsia temminckii*	KP306516.1	[58]
Black-Bellied Pangolin	*Phataginus tetradactyla*	MF509825	Current Study
White-Bellied Pangolin	*Phataginus tricuspis*	MF536683	Current Study
Giant Ground Pangolin	*Smutsia gigantea*	MF536684	Current Study
Temminck’s Ground Pangolin	*Smutsia temminckii*	MF536685	Current Study
Temminck’s Ground Pangolin	*Smutsia temminckii*	MF536686	Current Study
Temminck’s Ground Pangolin	*Smutsia temminckii*	MF536687	Current Study

The common name, scientific name, Genbank accession number and reference were noted for each individual. ^a^= Misidentified Chinese pangolin genome; ^b^= Misidentified Black-bellied pangolin genome

### Codon usage analysis for African and Asian species

The Relative Synonymous Codon Usage (RSCU) values for mitochondrial genes were established using the Mega v7 [88] software. This was performed on the four African pangolin species evaluated in this study as well as for the previously published *M. pentadactyla* (KT445978.1) and *M. javanica* (KT445979.1). The Principle Component Analysis (PCAs) generated from this data was performed using the FactoMineR package in R [89]. Codon usage bias (AT3 and GC3 content) was calculated using Mega v7, for each of the protein coding genes, where the A and T values at the third base were summed for the AT3 value. The same was performed with G and C for the GC3 content. The ratios were reported as percentages.

### Confirmation of insertions observed in the control region of the pangolin mitogenome

Sanger sequencing of the control region of the mitochondrial genome was performed using five additional samples from each of the African pangolin species. The white-bellied and black-bellied pangolin samples were from Ghana and the Temminck’s ground pangolin samples from South Africa and Tanzania [58]. The giant ground pangolin samples were obtained from the collection of the Zoological Museum, University of Copenhagen. The protocol and cycle conditions outlined in Du Toit [56] were used for all the samples. Sequencing was conducted in order to verify the presence of insertions in the D-loop observed in the mitogenomes obtained from next-generation sequencing during this study. A sequence fragment of around 500 bp was targeted using the primer pair: PNG_Dloop forward 5′-CGTTCCTCTTAAATAAGACATCTCG-3′ and reverse 5′-TCTTGCTTTTGGGGTTTGAC-3′ for verification.

## Results and discussion

### Next-generation sequencing

The HiSeq rapid run resulted in approximately 22 million reads per sample, with an average read length of 250 nucleotides. These reads were used for a De Novo assembly of each sample (CLC Bio version 6.0). This resulted in 10,207 contigs for *P. tricuspis* with the largest contig being 16,565 nucleotides, consisting of 78,099 reads at an average coverage of 986×. For *P. tetradactyla*, there were 2801 contigs with the largest contig, 16,649 nucleotides consisting of 47,686 reads at an average coverage of 369×. For *S. gigantea*, there were 11,346 contigs with the largest contig, 16,540 nucleotides consisting of 13,076 reads and an average coverage of 98×. For the three *S. temminckii* samples (MF536685–MF536687), contigs ranged from 2742 to 5560. The largest contig in all three samples was 16,558 nucleotides consisting of 63,759; 29,702 and 6820 reads and an average coverage of 494×; 248× and 53× respectively. The contigs were identified as the mitogenomes of the pangolin species based on the (i) estimated length (≈16.5 kb); (ii) the occurrence of the proteins *CoxI*, *Cob*, NADH dehydrogenase V (*NadV*) and NADH dehydrogenase VI (*NadVI*) (Table 2) and (iii) correspondence with mitochondrial sequences from other Pholidota based on NCBI BLAST searches.

**Table 2:** List of mitochondrial genes and loci, indicating size in base pairs from four African pangolin species, *Smutsia gigantea*, *S. temminckii*, *Phataginus tricuspis* and *P. tetradactyla*

Gene Regions	*S. gigantea* (Giant ground pangolin)	*S. temminckii* (Temminck’s ground pangolin)	*P. tricuspis* (White-bellied pangolin)	*P. tetradactyla* (Black-bellied pangolin)
Mitogenome (bp)	16,540	16,558	16,565	16,649
12S Ribosomal RNA (rRNA)	960	959	958	958
16S Ribosomal RNA (rRNA)	1560	1556	1555	1561
NADH dehydrogenase I (*NadI*)	951	945	945	945
NADH dehydrogenase II (*NadII*)	1038	1038	1038	1038
Cytochrome c oxidase I (*CoxI*)	1536	1533	1536	1515
Cytochrome c oxidase II (*CoxII*)	681	681	681	681
ATP synthase VIII (*AtpVIII*)	195	195	198	192
ATP synthase VI (*AtpVI*)	675	675	675	675
Cytochrome c oxidase III (*CoxIII*)	783	783	783	783
NADH dehydrogenase III (*NadIII*)	345	345	345	345
NADH dehydrogenase IV-L (*NadIV*-L)	294	294	294	294
NADH dehydrogenase IV (*NadIV*)	1371	1368	1371	1368
NADH dehydrogenase V (*NadV*)	1791	1788	1794	1803
NADH dehydrogenase VI (*NadVI*)	519	516	519	519
Cytochrome b (*Cob*)	1134	1134	1134	1134
Control region (D-loop)	1135	1155	1167	1265

### Genomic organisation

**Fig. 1:**
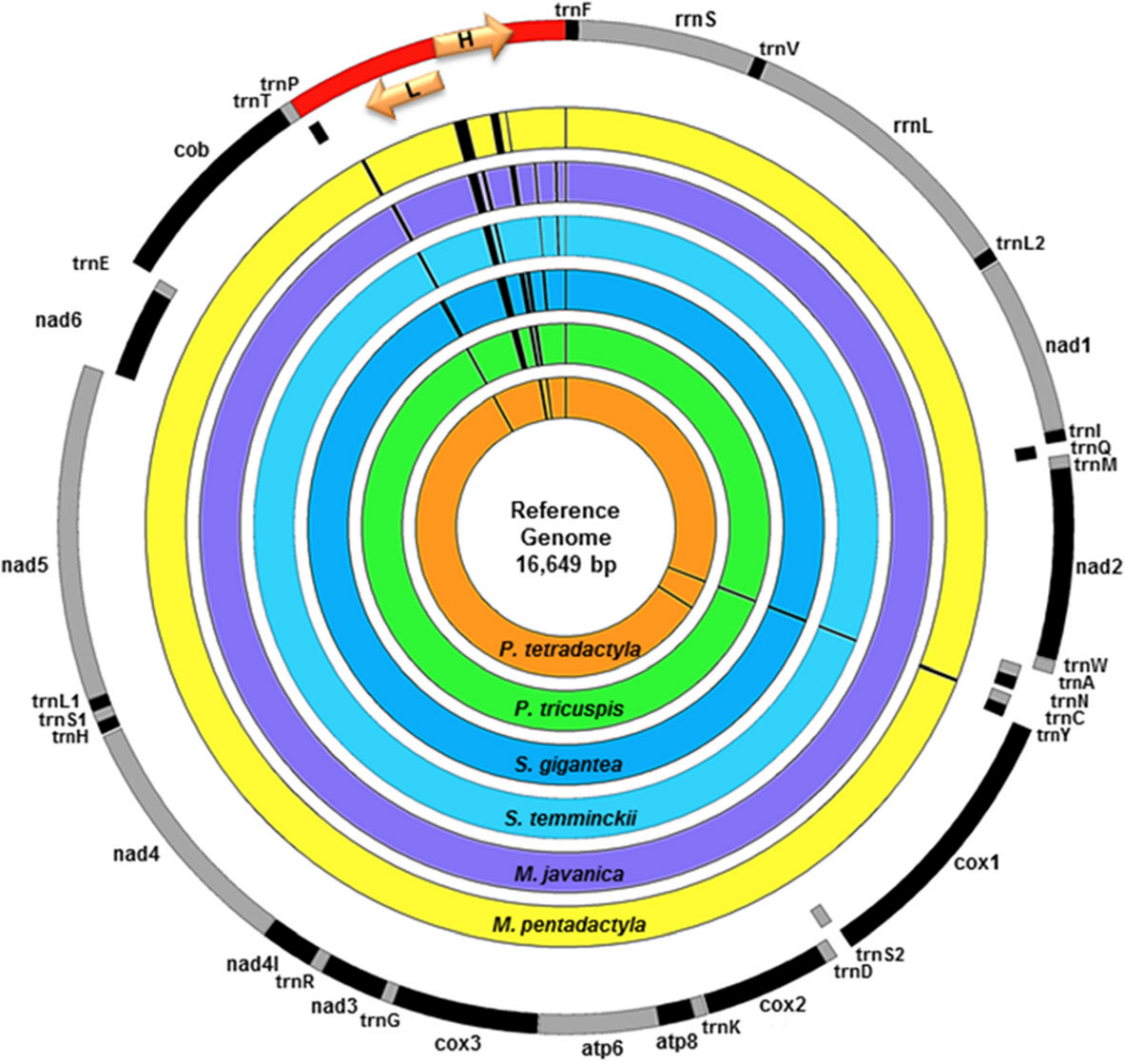
Circular diagram of six pangolin mitogenomes. The six coloured circles represent the six different pangolin species (four African and two Asian) aligned to each other to indicate the differences between individuals with the reference genome represented by *P. tetradactyla*. The outer ring represents the annotated loci located on the plus/heavy strand. The second ring represents the loci located on the minus/light strand of the mitogenome. Arrows are representative of the direction of the light and heavy strands; the heavy strand is located clockwise and the light strand anti-clockwise

The mitogenome of the *S. temminckii* samples consisted of 16,558 bp while *S. gigantea* was 16,540 bp; *P. tetradactyla* was 16,649 bp and *P. tricuspis* was 16,565 bp in length (Table 2, Fig. 1). The light and heavy strands each contain their own arrangement of genes, proteins or loci respectfully located on each strand (Fig. 1). The heavy strand, or plus strand, comprises of the following loci: two Ribosomal RNAs (12S rRNA, 16S rRNA); 12 Protein-coding genes (*NadI, NadII*, *CoxI*, *CoxII*, *AtpVIII*, *AtpVI*, *CoxIII*, *NdIII, NadIV-L*, *NadIV*, *NadV*, *Cob*) and 14 Transfer RNAs (trnF, trnV, trnL2, trnI, trnM, trnW, trnD, trnK, trnG, trnR, trnH, trnS1; trnL1, trnT). The light or minus strand comprises one Protein-coding gene (*NadVI*) and eight Transfer RNAs (trnQ, trnA, trnN, trnC, trnY, trnS2, trnE, trnP). As indicated in Table 2, the mitogenome of the four African pangolins varied in terms of gene region size at several loci. Six regions [cytochrome oxidase II (*CoxII*), ATP synthase VI (*AtpVI*), cytochrome oxidase III (*CoxIII*), NADH dehydrogenase III (*NadIII*), NADH dehydrogenase IV-L (*NadIV-L*), cytochrome b (*COB*)] were found to be the same length in all four species and three loci [16S Ribosomal RNA, NADH dehydrogenase V (*NadV*), D-loop] each have different lengths in each of the four species. The remaining loci [12S Ribosomal RNA, NADH dehydrogenase I (*NadI*), NADH dehydrogenase II (*NadII*), cytochrome oxidase I (*CoxI*), ATP synthase VIII (*AtpVIII*), NADH dehydrogenase IV (*NadIV*), NADH dehydrogenase VI (*NadVI*)] had lengths that were generally consistent, with some pangolin species showing variation in length in comparison to the other species (Table 2).

### Phylogenetic analysis of African pangolins

**Fig. 2:**
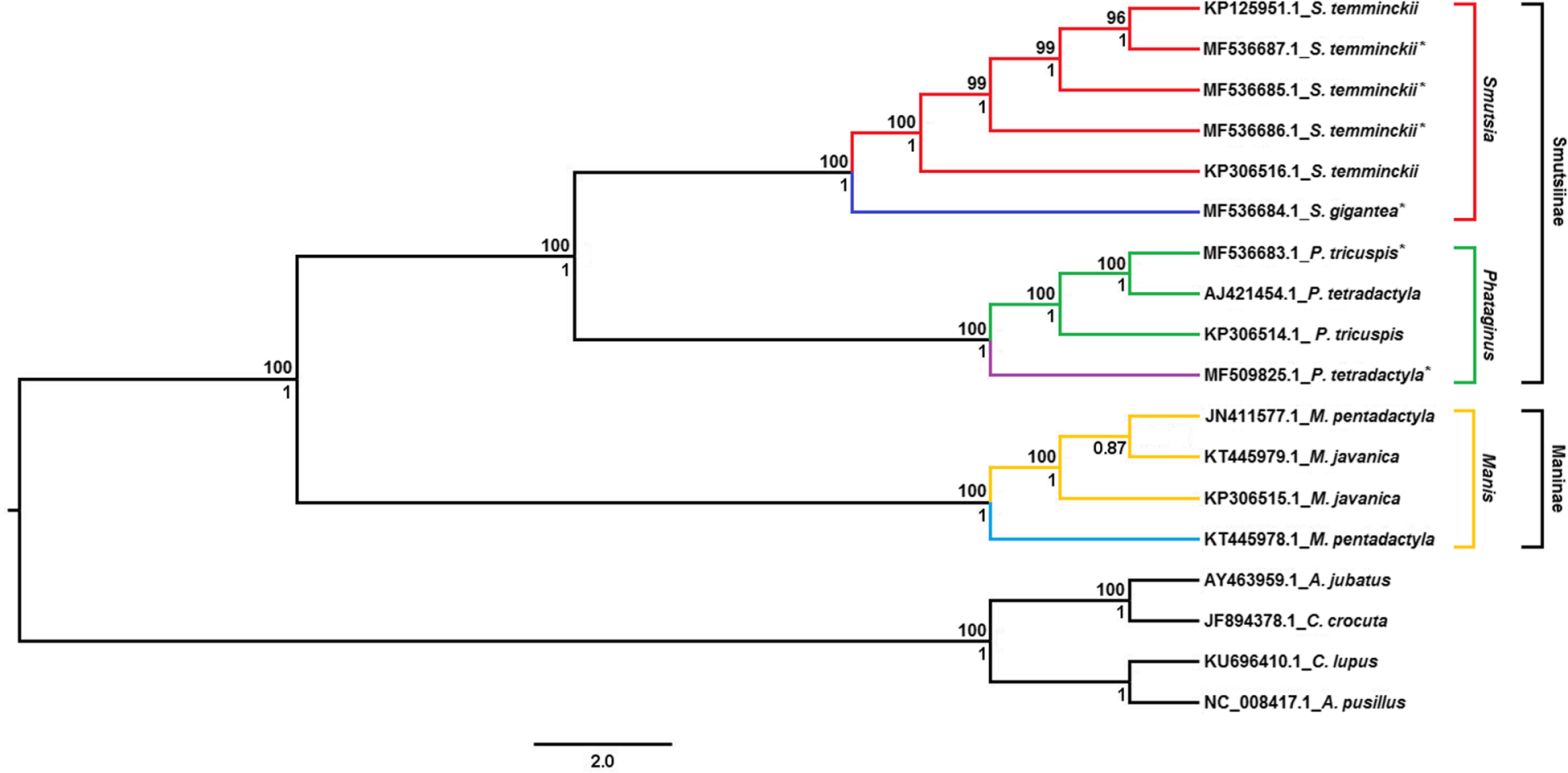
Combined Bayesian Inference (BI) and Maximum Likelihood (ML) tree of pangolin species. Bayesian Posterior Probabilities are indicated on the bottom of each node whereas the Maximum Likelihood Bootstrap values are indicated on top of the node. Only Bootstrap values equal or greater than 70% (≥70%) were noted on the tree. The mitogenome of *Acinonyx. jubatus*, *Crocuta crocuta*, *Canis lupus* and *Arctocephalus pusillus* was selected as outgroups. * indicates the six mitogenomes sequenced during this study

The best fit model of sequence evolution for the dataset under the AIC was the General Time Reversal model (GTR + I + G) with invariable site and gamma distribution of 0.822 [90, 91]. The best fit model under the BIC and DT was the Transition model two (TIM2 + I + G) with invariable site and gamma distribution values of 0.998 and 0.990 respectively [92]. From these two models three Bayesian phylogenetic trees along with three maximum likelihood trees were generated for the datasets. The different trees all showed consistent branching patterns, posterior probability values for the BI trees and bootstrap values for the ML trees. The trees were subsequently concatenated into a single consensus tree with the different support values indicated on the respective branches (Fig. 2). Partition analysis indicated a variety of models for the individual loci and was subjected to individual BI and ML analysis to confirm the results of the whole mitochondrial data. In the phylogenetic tree, using all available pangolin mitogenomes, it is evident that all the African pangolins group according to species, with the exception of the black-bellied pangolin genome (AJ421454.1) [54]. The latter mitogenome has previously been reported to be misclassified based on partial *Cob* analysis (Additional file 1: Figure S1) [58, 60] and was confirmed in this analysis as a white-bellied pangolin genome. In addition, the misclassified *M. pentadactyla* (JN411577.1) [55] grouped with *M. javanica* [58, 59], confirming an error also reported in previous studies. The phylogenetic tree (Fig. 2) which excluded the misclassified samples provided support for the Asian and African pangolin species separation into two distinct monophyletic clades with the latter consisting of all African pangolins species, *P. tricuspis, P. tetradactyla, S. temminckii* and *S. gigantea*. Within the African clade the giant and Temminck’s pangolin clustered separately from the white-bellied and black-bellied pangolins with significant Bayesian and ML support (Posterior Probabilities of 1). For the African pangolin species, the observed branching pattern thus provides support for the classification of the ground-dwelling and arboreal species into two separate genera; *Phataginus* and *Smutsia*. In addition, results from this analysis suggests the overall classification of pangolin into three genera; *Manis* (Asian pangolins), *Phataginus* (African tree pangolins) and *Smutsia* (African ground pangolins). However, further analysis should be undertaken for Asian pangolins to include the full mitochondrial genomes of the Philippine (*M. culionensis*) and Indian pangolin (*M. crassicaudata*). The above branching patterns were also confirmed using individual loci. The control region, rRNAs, light strand proteins, light strand tRNAs and heavy strand proteins (exclusive of COX2) BI and ML results were all concurrent with the whole mtDNA tree with high support. The heavy strand tRNAs showed differentiation in the tree pangolins and again in the ground pangolins of Africa for both BI and ML trees. Although the internal grouping differs, they still conform to the same three genera identified in the trees above namely *Smutsia*, *Phataginus* and *Manis*. The ML analysis for the heavy strand protein COX2 was in accordance with the results above. However, the BI analysis indicated that the black-bellied pangolin (MF509825) branched separately from the African pangolins, but still formed a monophyletic group with the African pangolin species adjacent to the Asian pangolins. Overall, the majority of the individual loci subject to different evolutionary rates and models along with a variety of phylogenetic analysis concurred with the results obtained from the whole mtDNA data.

### Analysis of GC content and codon usage

Total GC content of the African pangolin species was observed to be similar between species (*P. tetradactyla* = 36.5%, *S. gigantea* = 36.9%, *S. temminckii* = 37.3% and *P. tricuspis* = 36.7%). These results confirm an AT-bias that has been reported in several other mammal species [93]. Analysis of codon usage and pattern of mitochondrial genes; *AtpVI*, *AtpVIII*, *Cob*, *CoxI*, *CoxII*, *NadI*, *NadII*, *NadIII*, *NadIV*, *NadIV-L*, *NadV* and *NadVI* provided evidence of bias in terms of the use of codons with A and C occurring most frequently at the third codon (Additional file 1: Table S1). Variation in base compositions within and among species has been suggested to occur as a result of two evolutionary processes namely biases in the process of mutation and/or natural selection [94]. Selective nucleotide compositional biases have been reported in chiropteran mitochondrial genomes [95]. Uddin and Chakraborty [96] similarly observed A or C as the most frequent codon at the 3rd position in a study of mitochondrial *AtpVI* in a variety of mammalian species. The authors attributed this bias to mutational pressure that can influence codon usage bias in mitochondria.

### GC content and codon usage variation between pangolin species

**Fig. 3:**
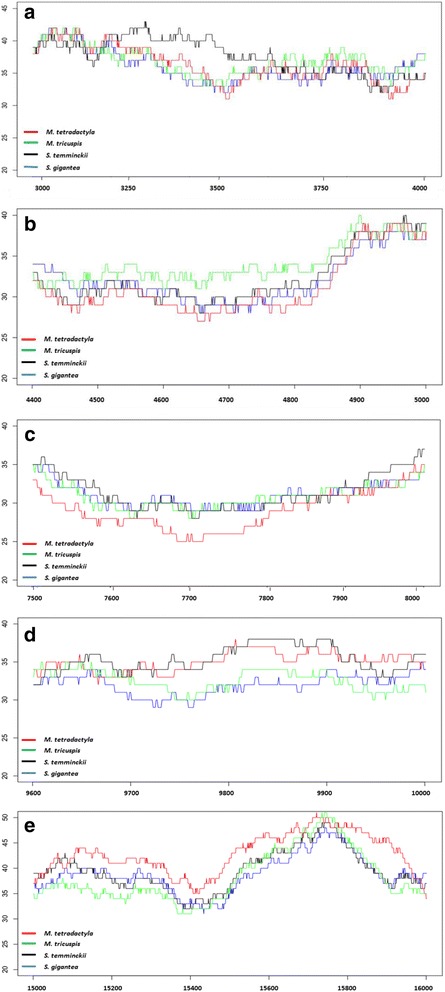
Representation of regions which display significant differences in terms of GC content among African pangolin species (**a**-**e**). Image (**a**) showing increased GC content (~3100–3700 bp) in *S. temminckii*; (**b**) showing an increased GC content (~4500–4900 bp) in *M. tricuspis*; (**c**) showing a decreased GC content (~7500–7900 bp) in *M. tetradactyla*; (**d**) showing an increased GC content (~9700–9900 bp) in *M. tetradactyla* and *S. temminckii*; (**e**) showing an increased GC content (~15,000–16,000 bp) in *M. tetradactyla*

**Fig. 4:**
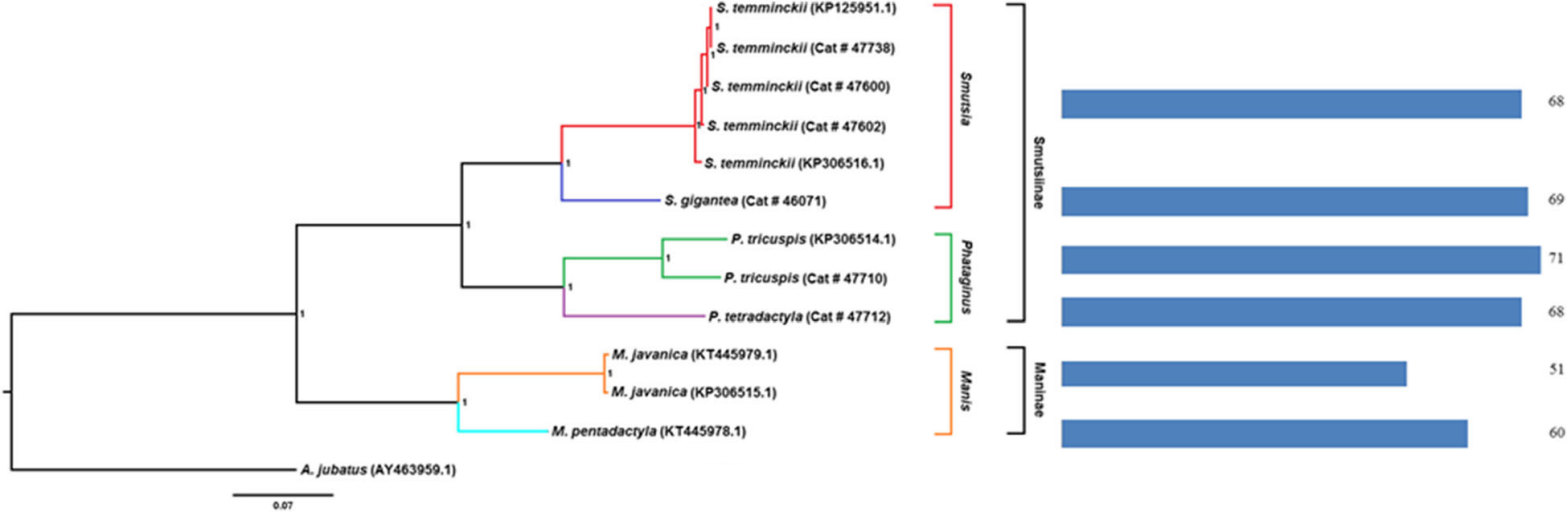
Clustering of pangolin species according to the variation of codon usage and phylogeny. The dendrogram was inferred by hierarchical clustering with the AT3 frequency metric for each species. The value of the AT3 is indicated as a percentage

The percentage of GC content and codon frequencies calculated for five regions (3100–3700 bp, 4500–4900 bp, 7500–7900 bp, 9700–9900 bp and 15,000–16,000 bp) of the mitogenome was significantly different between the four African pangolin species based on ANOVA analysis (Fig. 3a -e). The respective genes that correspond to these regions include *NadI*, *NadII*, *CoxII*, *NadIII* and *Cob*. Analysis of codon usage of three mitochondrial genes; *CoxI*, *NadI* and *NadIII* reveals a clear distinction between the African and Asian pangolins, as well as within the African clade (Additional file 1: Figure S2 a-c). The PCA plots for these three genes therefore identified a connection between codon usage and phylogeny and provide further support for the phylogenetic analysis based on the whole mitochondrial genome. The PCA plots of the remaining protein coding genes (*AtpVIII*, *NadIV* and *NadVI*), whilst not achieving the same resolution within the African clade, show a clear separation of the Asian and African pangolins (Additional file 1: Figure S2 d-f). Combined codon usage patterns of mitochondrial genes (*AtpVI*, *AtpVIII*, *Cob*, *CoxI*, *CoxII*, *NadI*, *NadII*, *NadIII*, *NadIV*, *NadIV-L*, *NadV* and *NadVI*) were plotted in order to perform a hierarchical clustering of each species to investigate the role of codon bias in the evolution of African pangolins. The resulting dendrogram is presented in Fig. 4. African pangolin species varied in the percentage of codon bias with Asian pangolins displaying a lower degree of AT3 bias. Variations in GC content and codon frequencies between pangolin species may indicate that two selective forces; mutational pressure and/or natural selection may play important roles in the molecular evolution of pangolins with different evolutionary forces acting to shape the mitochondrial genomes of the African and Asian pangolin species.

### Mitogenome comparison between *Smutsia* and *Phataginus*

**Fig. 5:**
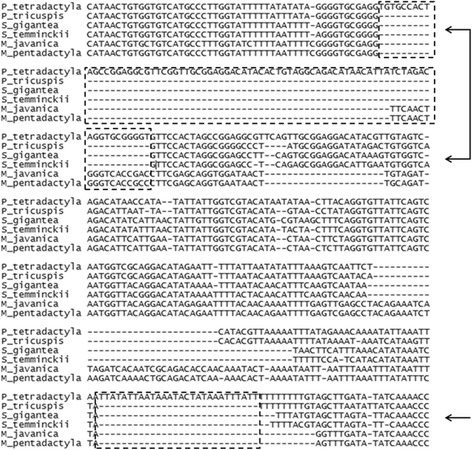
Alignment of a region of D-loop in six pangolin species. The insertion sequence for *P. tetradactyla* is indicated in a dashed box. The first insertion is 80 bp and the second 28 bp in length

Two insertions (80 bp and 28 bp in length) in the D-loop region of the black-bellied pangolin were observed (Fig. 5) which was absent in the other African pangolin species. This insertion was validated with Sanger sequencing that included additional African pangolin species. When compared to the Asian pangolin species the first insertion was slightly shorter in length (62 bp). Length variation in the D-loop has been reported in various species including bats [97], rodents [98] and primates [99]. In addition, substantial nucleotide sequence differences within and between species have been identified in the D-loop [100, 101]. Lastly, heteroplasmy where individuals had more than one mtDNA form due to variation in numbers of tandem repeats in D-loop has been reported in shad [102], sturgeon [103], whiptail lizards [104] and rabbits [105]. Length variation has been proposed to occur via four different mechanisms including illegitimate elongation [103], intra- and intermolecular recombination [106], transposition [106] and slipped miss pairing [107]. The identification of the two insertions in the D-loop region may indicate that this region is under selection in black-bellied pangolins, which may demonstrate drift following the initial mutation event. However, further analysis among closely related species should be conducted in order to determine how selection impacts on the length and sequence variation within this region.

## Conclusions

In conclusion, our research study presents the mitogenomes for the four African pangolin species. These include two new reference genomes for the black-bellied pangolin and the giant ground pangolin. This study also presents the first phylogenetic assessment of six of the eight extant pangolin species using whole mitochondrial DNA genomes. The African and Asian pangolin species are shown to separate into two distinct monophyletic clades. Within the African pangolins it was further demonstrated that there is support for classification of the species into separate genera, representing the arboreal (*P. tricuspis*, *P. tetradactyla*) and ground-dwelling (*S. temminckii* and *S. gigantea*). The availability of these reference mitogenomes will, furthermore, contribute to a better understanding of the evolutionary processes of pangolin species globally, which in turn can contribute to essential conservation genetic studies.

## Additional files

Additional file 1: Figure S1. Bayesian mitogenome phylogenetic tree of all available pangolin mitogenomes. Posterior probabilities are indicated on the respective branches. *A. jubatus* was selected as an outgroup as pangolins are more closely related to the order Carnivora. Asterisks indicate the misidentified mitogenomes. **Figure S2.** Principal Component Analysis (PCA) of Relative Synonymous Codon Usage values (RSCU) for six pangolin species. Three distinct genera is present (*Manis*, *Phataginus* and *Smutsia*) in the (a) *CoxII*; (b) *NadI*, (c) *NadIII* genes. The two sub-families (Smutsiinae and Maninae) are distinguished in the (d) *AtpVIII*; (e) *NadIV*; (f) *NadVI* genes. **Table S1.** List of nucleotide percentages and its 3rd codon position percentage (%) (PDF 426 kb)
