# Supplementary material for: Mitochondrial genomes of African pangolins and insights into evolutionary patterns and phylogeny of the family Manidae
Source: BMC Genomics. 2017 Sep 21;18:746. doi: 10.1186/s12864-017-4140-5 (PMC5609056; doi:10.1186/s12864-017-4140-5)
Supplement: Supplementary file 1 — Bayesian mitogenome phylogenetic tree of all available pangolin mitogenomes. Posterior probabilities are indicated on the respective branches. A. jubatus was selected as an outgroup as pangolins are more closely related to the order Carnivora. Asterisks indicate the misidentified mitogenomes. Figure S2. Principal Component Analysis (PCA) of Relative Synonymous Codon Usage values (RSCU) for six pangolin species. Three distinct genera is present (Manis, Phataginus and Smutsia) in the (a) CoxII; (b) NadI, (c) NadIII genes. The two sub-families (Smutsiinae and Maninae) are distinguished in the (d) AtpVIII; (e) NadIV; (f) NadVI genes. Table S1. List of nucleotide percentages and its 3rd codon position percentage (%) (PDF 426 kb) [file 12864_2017_4140_MOESM1_ESM.pdf]

Mitochondrial genomes of African pangolins and insights into evolutionary patterns and phylogeny of the family Manidae

Zelda du Toit, Morné du Plessis, Desiré L. Dalton*, Raymond Jansen, J. Paul Grobler and Antoinette Kotzé

*Corresponding Author, e-mail: desire@nzg.ac.za , (Tel) +27 12 328 3265

Additional Information Figures and Table

Fig. S1: Bayesian mitogenome phylogenetic tree of all available pangolin mitogenomes. Posterior probabilities are indicated on the respective branches. *A. jubatus* was selected as an outgroup as pangolins are more closely related to the order Carnivora. Asterisks indicate the misidentified mitogenomes.

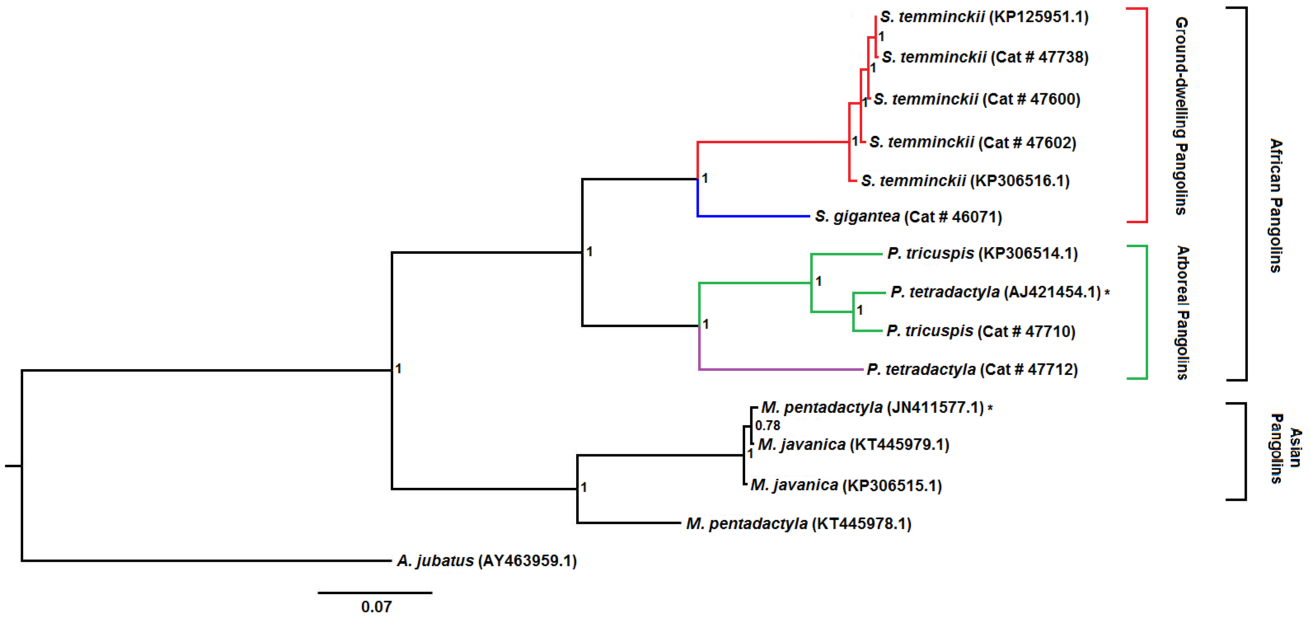

Fig. S2: Principal Component Analysis (PCA) of Relative Synonymous Codon Usage values (RSCU) for six pangolin species. Three distinct genera is present (*Manis*, *Phataginus* and *Smutsia*) in the (a) *CoxII*; (b) *NadI*, (c) *NadIII* genes. The two sub-families (Smutsiinae and Maninae) are distinguished in the (d) *AtpVIII*; (e) *NadIV*; (f) *NadVI* genes.

(a)

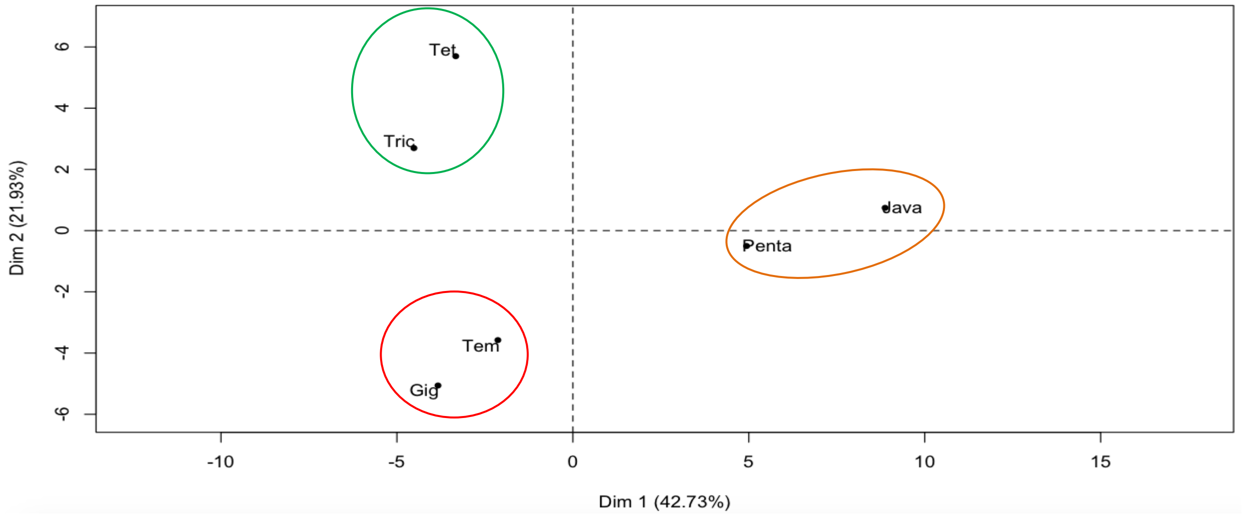

(b)

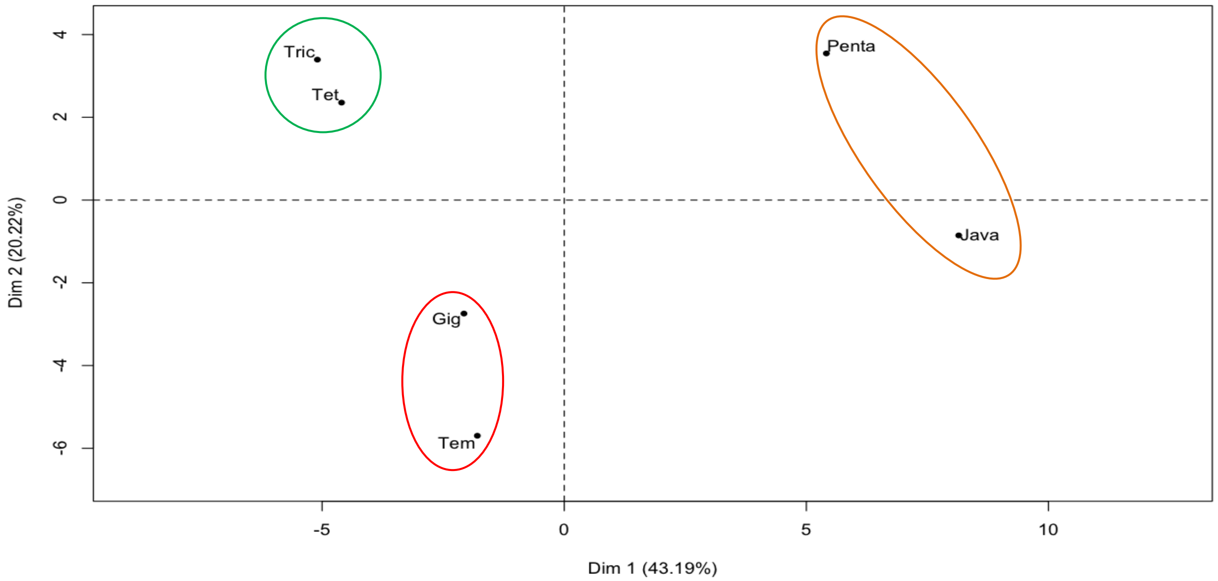

(c)

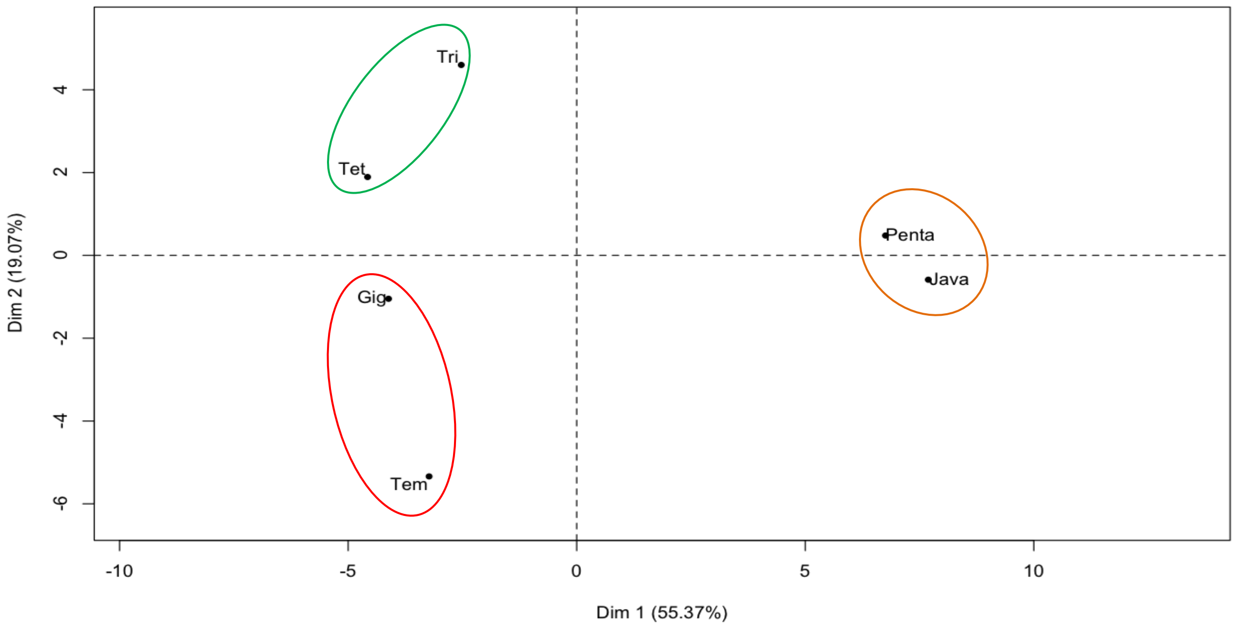

Tri
Tet
Penta
Java
Gig
Tem
Dim 2 (19.07%)
Dim 1 (55.37%)

(d)

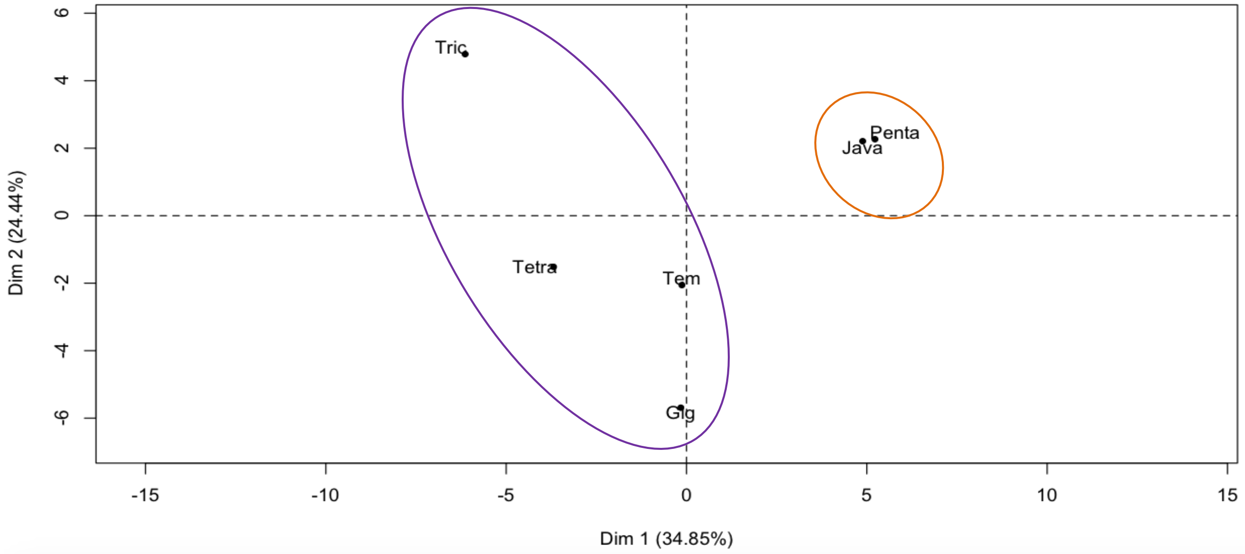

Tric
Penta
Java
Tetra
Tem
Gig
Dim 2 (24.44%)
Dim 1 (34.85%)

(e)

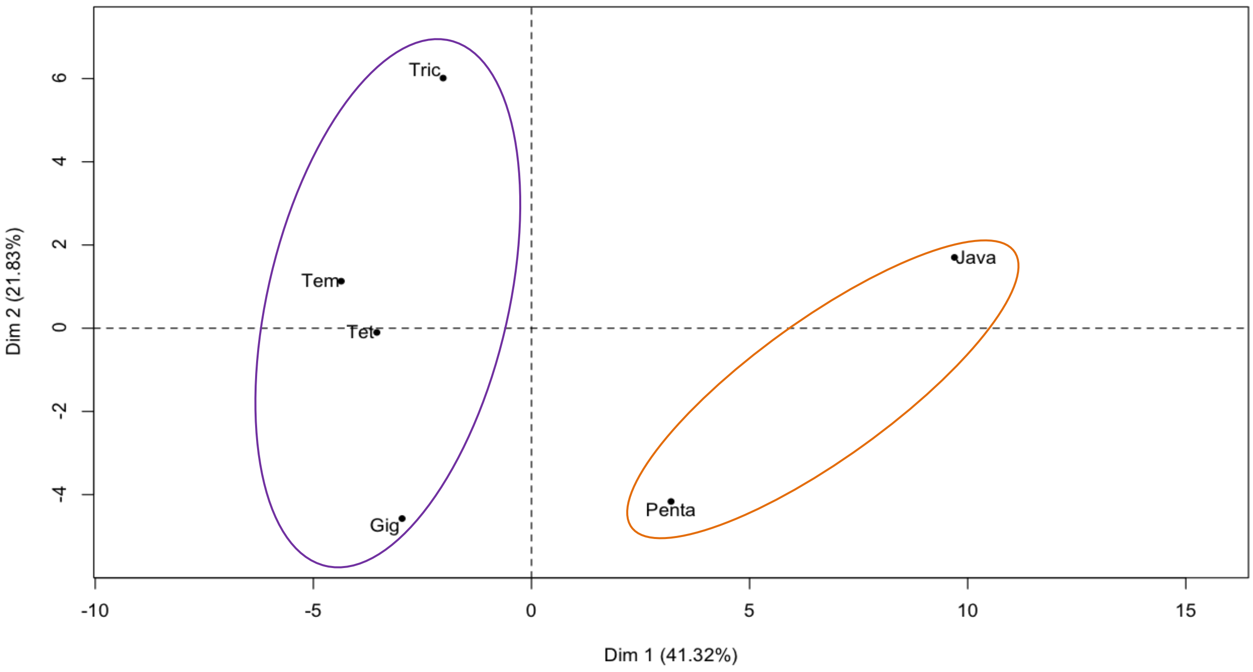
Tric
Tem
Tet
Gig
Java
Penta
Dim 2 (21.83%)
Dim 1 (41.32%)
6
4
2
0
-2
-4
-10
-5
0
5
10
15

(f)

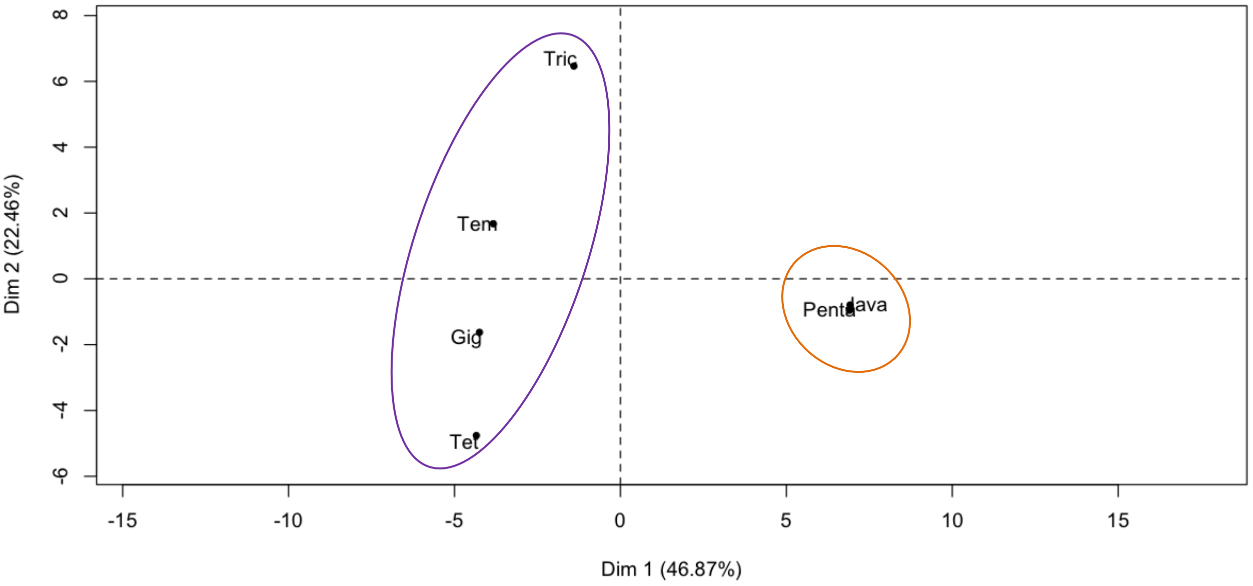
Tric
Tem
Gig
Tet
Penta
Java
Dim 2 (22.46%)
Dim 1 (46.87%)
8
6
4
2
0
-2
-4
-6
-15
-10
-5
0
5
10
15

Table S1: List of nucleotide percentages and its 3rd codon position percentage (%).

Species	% of A	A3%	% of T	T3%	% of G	G3%	% of C	C3%
**ATP6**								
M. pentadactyla	34.4	46.7	26.4	20	9.8	2.6	29.4	30.8
M. javanica	31.6	38.3	23.6	12	11.9	8.8	32.9	41.0
P. tricuspis	32.4	40.4	31.7	32	10.7	6.2	25.2	20.9
P. tetradactyla	33.9	44.0	31.1	30	9.5	3.6	25.5	22.7
S. gigantea	33.3	44.0	29.3	25	9.5	3.1	27.9	27.6
S. temminckii	32.0	40.9	29.5	26	10.8	5.8	27.7	27.1
**ATP8**								
M. pentadactyla	38.8	43.3	23.9	16	7.5	9.0	29.9	31.3
M. javanica	38.7	45.6	22.5	13	7.8	8.8	30.9	32.4
P. tricuspis	35.9	39.4	33.3	41	6.1	6.1	24.7	13.6
P. tetradactyla	39.1	43.8	34.4	39	5.7	4.7	20.8	12.5
S. gigantea	37.4	41.5	31.8	29	5.6	4.6	25.1	24.6
S. temminckii	39.5	47.7	30.3	28	5.6	4.6	24.6	20.0
**Cytb**								
M. pentadactyla	30.9	42.9	25.6	14	13.4	3.4	30.1	40.0
M. javanica	30.0	42.3	24.4	10	14.2	5.6	31.4	42.1
P. tricuspis	30.9	41.5	31.1	26	13.1	3.2	25.0	29.6
P. tetradactyla	31.2	42.1	29.5	23	12.9	3.4	26.4	31.5
S. gigantea	32.5	44.7	28.0	18	12.2	2.4	27.3	34.9
S. temminckii	31.7	43.4	28.3	19	12.4	2.9	27.5	34.7
**COI**								
M. pentadactyla	28.4	39.3	27.9	21	16.6	5.8	27.1	33.7
M. javanica	26.1	32.8	26.6	18	18.6	11.4	28.6	38.0
P. tricuspis	27.0	26.8	34.1	26	16.8	29.2	22.0	18.4
P. tetradactyla	27.9	38.0	33.4	33	15.7	4.4	23.0	25.0
S. gigantea	29.0	41.8	32.1	31	15.8	3.3	23.0	24.2
S. temminckii	27.7	39.3	33.3	35	16.8	4.7	22.2	21.3
**COII**								
M. pentadactyla	32.6	41.7	24.9	17	13.5	6.1	29.1	35.5
M. javanica	31.3	37.3	21.9	11	14.8	10.1	32.0	42.1
P. tricuspis	32.6	42.7	30.8	31	13.4	5.7	23.2	20.7
P. tetradactyla	33.0	44.1	30.2	28	13.7	6.6	23.1	21.1
S. gigantea	33.9	46.7	29.2	28	12.3	2.6	24.5	22.5
S. temminckii	33.2	42.7	29.2	27	12.9	5.7	24.7	24.2
**COIII**								
M. pentadactyla	29.3	42.5	26.8	17	14.5	3.4	29.3	36.8
M. javanica	27.8	39.1	24.4	11	15.8	6.9	32.0	42.9
P. tricuspis	26.6	35.6	34.1	33	15.6	5.7	23.8	25.3
P. tetradactyla	28.2	39.1	33.2	30	13.8	1.9	24.8	29.1
S. gigantea	29.6	44.1	29.8	24	13.9	1.5	26.7	30.3

S. temminckii	28.1	37.5	31.7	27	14.9	6.5	25.3	29.1
**ND1**								
M. pentadactyla	32.8	52.8	24.8	11	12.3	3.1	30.0	33.0
M. javanica	30.3	45.9	22.6	5	14.9	10.1	32.2	38.7
P. tricuspis	31.0	46.3	30.9	26	13.1	5.4	25.0	22.2
P. tetradactyla	31.4	48.6	29.0	23	13.4	6.0	26.1	22.9
S. gigantea	33.0	51.4	29.0	21	11.7	2.2	26.3	24.9
S. temminckii	31.9	47.6	28.0	20	13.4	7.3	26.7	25.1
**ND2**								
M. pentadactyla	37.3	51.9	24.3	13	8.9	2.9	29.5	32.5
M. javanica	37.1	52.3	21.2	5	10.5	6.4	31.3	36.1
P. tricuspis	35.9	50.0	28.6	18	10.3	6.4	25.1	25.4
P. tetradactyla	38.2	54.9	29.5	23	9.1	2.9	23.3	19.7
S. gigantea	38.9	55.8	27.2	15	7.9	1.7	26.0	27.5
S. temminckii	38.3	54.3	28.3	18	8.0	2.6	25.3	24.6
**ND3**								
M. pentadactyla	34.6	53.9	25.1	9	11.0	3.5	29.4	33.9
M. javanica	31.5	45.2	22.0	3	13.9	10.4	32.7	41.7
P. tricuspis	31.9	51.3	31.6	20	11.3	3.5	25.2	25.2
P. tetradactyla	32.8	52.2	32.5	24	9.9	.9	24.9	22.6
S. gigantea	34.1	54.4	30.9	20	9.6	.9	25.4	24.6
S. temminckii	31.9	48.7	33.0	24	11.9	6.1	23.2	20.9
**ND4**								
M. pentadactyla	34.4	47.7	25.5	15	10.6	2.8	29.5	34.4
M. javanica	32.4	44.0	21.9	6	12.6	6.8	33.0	43.1
P. tricuspis	33.0	44.2	30.1	24	11.6	5.7	25.3	26.0
P. tetradactyla	33.7	45.6	30.5	28	10.3	3.3	25.5	23.5
S. gigantea	34.1	47.7	28.8	22	10.1	2.2	27.1	28.2
S. temminckii	34.0	45.8	29.2	26	10.7	4.6	26.0	23.9
**ND4L**								
M. pentadactyla	32.3	47.5	26.9	8	11.4	6.1	29.3	32.3
M. javanica	30.6	41.4	26.3	10	12.5	10.1	30.6	30.6
P. tricuspis	29.3	37.8	36.1	33	11.2	5.1	23.5	29.3
P. tetradactyla	27.2	34.7	35.0	30	12.6	6.1	25.2	27.2
S. gigantea	30.6	44.9	36.4	31	10.2	1.0	22.8	30.6
S. temminckii	28.9	40.8	32.0	26	12.6	5.1	26.5	28.9
**ND5**								
M. pentadactyla	34.9	44.6	24.8	13	9.8	1.3	30.4	40.9
M. javanica	33.6	42.2	20.9	6	11.5	4.8	34.0	47.4
P. tricuspis	34.1	44.8	29.9	24	11.4	4.3	24.5	26.8
P. tetradactyla	35.1	46.1	30.1	24	10.5	2.7	24.3	26.8
S. gigantea	35.0	45.7	27.3	19	10.1	1.7	27.7	34.0
S. temminckii	33.8	43.8	28.4	21	10.9	3.2	26.9	32.0

ND6								
M. pentadactyla	45.3	30.3	17.5	21	6.5	4.0	30.7	44.6
M. javanica	43.8	27.4	13.7	19	9.0	6.9	33.5	46.3
P. tricuspis	42.0	26.6	21.4	25	8.1	5.8	28.5	42.2
P. tetradactyla	41.8	26.0	22.0	25	6.4	5.2	29.9	43.4
S. gigantea	45.1	30.1	19.5	24	6.4	3.5	29.1	42.8
S. temminckii	43.4	30.2	20.5	24	7.2	3.5	28.9	42.4
